# Role of macrophages in pulmonary arterial hypertension

**DOI:** 10.3389/fimmu.2023.1152881

**Published:** 2023-04-19

**Authors:** Meng-Qi Zhang, Chen-Chen Wang, Xiao-Bin Pang, Jun-Zhuo Shi, Hao-Ran Li, Xin-Mei Xie, Zhe Wang, Hong-Da Zhang, Yun-Feng Zhou, Ji-Wang Chen, Zhi-Yan Han, Lu-Ling Zhao, Yang-Yang He

**Affiliations:** ^1^ School of Pharmacy, Henan University, Kaifeng, Henan, China; ^2^ State Key Laboratory of Cardiovascular Disease, Fuwai Hospital, National Center for Cardiovascular Diseases, Chinese Academy of Medical Sciences and Peking Union Medical College, Beijing, China; ^3^ Department of Medicine, University of Illinois at Chicago, Chicago, IL, United States

**Keywords:** Pulmonary arterial hypertension, macrophages, inflammatory response, vascular remodeling, macrophage microenvironment

## Abstract

Pulmonary arterial hypertension (PAH) is a severe cardiopulmonary vascular disease characterized by progressive pulmonary artery pressure elevation, increased pulmonary vascular resistance and ultimately right heart failure. Studies have demonstrated the involvement of multiple immune cells in the development of PAH in patients with PAH and in experimental PAH. Among them, macrophages, as the predominant inflammatory cells infiltrating around PAH lesions, play a crucial role in exacerbating pulmonary vascular remodeling in PAH. Macrophages are generally polarized into (classic) M1 and (alternative) M2 phenotypes, they accelerate the process of PAH by secreting various chemokines and growth factors (CX3CR1, PDGF). In this review we summarize the mechanisms of immune cell action in PAH, as well as the key factors that regulate the polarization of macrophages in different directions and their functional changes after polarization. We also summarize the effects of different microenvironments on macrophages in PAH. The insight into the interactions between macrophages and other cells, chemokines and growth factors may provide important clues for the development of new, safe and effective immune-targeted therapies for PAH.

## Highlights

• Macrophages play an important role in the PAH process, and imbalance of M1/M2 ratio is a significant feature of aggravating PAH.

• Macrophage recruitment in the perivascular area as a marker of inflammatory response initiation will be a key factor in exacerbating pulmonary vascular remodeling.

• Cytokines such as CCR5 and IL-1R1 expressed by both macrophages and PASMCs can be bi-directionally chemotactic and stimulate each other, cyclically aggravating the abnormal proliferation of PASMCs.

• Metabolic disorders and immune cell interactions as well as viral invasion can lead to alterations in the microenvironment of macrophages.

## 1 Introduction

Pulmonary arterial hypertension (PAH) is defined as mean pulmonary arterial pressure (mPAP) ≥20 mmHg during right heart catheterization at rest (1). PAH is a chronic progressive cardiovascular disease caused by the remodeling of pulmonary vascular structure and progressive pulmonary artery obstruction (2), which continuously increases pulmonary artery pressure, and can lead to right heart failure and even death in severe cases. The pathogenesis of PAH is complex and unclear. In addition to gene mutations, imbalance of vasoactive substances, immune inflammatory reaction and abnormal energy metabolism are also involved in the development of PAH (3). At present, most of the drugs on the market are for symptomatic treatments (4). Although the short-term survival rate of patients has been improved, there is still no cure. PAH has become a public health problem that endangers the society, and heavily burdens members of our community and the medical industry. This is attributable to the unclear pathogenesis of PAH (5), so in-depth studies on its course are fundamentally important for us to develop novel therapeutic strategies.

The pathology of PAH is characterized by irreversible tissue changes called “pulmonary vascular remodeling” involving pulmonary artery endothelial cells (ECs), smooth muscle cells (SMCs), and fibroblasts (6). There is increasing evidence that perivascular inflammation plays a functional role in pulmonary vascular remodeling. It has been found that a large number of immune cells such as macrophages, neutrophils, dendritic cells, mast cells, T lymphocytes and B lymphocytes were clustered around the pulmonary vessels in patients with PAH (7). Among them, several subtypes of macrophages play key roles in PAH progression. M1-type macrophages amplify inflammation by secreting pro-inflammatory factors, while M2-type macrophages promote tissue repair and play a major role in pulmonary vascular remodeling (8). Simultaneously, the expression levels of pro-inflammatory factors such as interleukin-1 beta (IL-1β), interleukin-6 (IL-6), interleukin-10 (IL-10), transforming growth factor beta (TGF-β) and tumor necrosis factor alpha (TNF-α) increased (9).

As an important part of the inflammatory process, macrophages are crucial in the development of PAH (10). In recent years, it is understood that macrophages can change their tissue remodeling by affecting cell survival, proliferation, migration and immune regulation (11). More and more evidence showed that the interaction between inflammatory cells, vascular cells and inflammatory mediators, which may provide an important theoretical basis for the development of new, safe and effective immune-targeted therapies for PAH.

Herein, this review is aimed to review the role of macrophages in the development of PAH.

## 2 Characteristics of macrophages and their pathophysiological significance

### 2.1 Origin and tissue distribution of macrophages

A complex host defense system that relies on innate immunity has evolved to contribute to the adaptation of environment and species diversity. Previous studies revealed that the source of macrophages was not the only one. Earlier scholars have found that phenotypically mature macrophages existed in some tissues before the emergence of hematopoietic stem cells based on the mouse model (12), and a subsequent study has shown that resident tissue macrophages (RTM) might emerge during the development of embryonic precursors (13). Experiments based on mouse model showed (14) that the replacement of embryonic liver monocytes by bone marrow-derived monocytes was not completed until 2 months after birth. Vascular macrophages, on the other hand, were more similar to dermal tissues and rapidly replaced by bone marrow-derived monocytes after birth (15, 16). In addition, calculations of monocyte influx into the spleen and local macrophages generated by DNA synthesis of monocyte phagocytes showed that in the steady-state mice, 55% of macrophages in the spleen were maintained by monocyte influx and 45% by local division of monocyte phagocytes. In addition, a quantitative study with analysis of macrophages in the rat ventricles of the brain revealed that the number of macrophages increased significantly with age and the increased number of macrophages in the cerebral ventricle was partly attributed to the proliferation of local cell, as mitotic cells were observed (17). Another explanation for the growing number of these cells was the uptake of blood monocytes and interstitial macrophages, which were thought to be their precursor cells (18). This implies that macrophages have a dual origin, that is, some macrophages are derived directly from circulating monocytes which are derived from bone marrow cells and others from tissue-resident, locally dividing mononuclear phagocytes.

Existing studies have shown that the tissue-resident macrophages were found to be divided into subpopulations based on their autopsy location and functioning phenotype, including microglia in the central nervous system, osteoclasts in bone, alveolar macrophages in the lung, phage cells in the spleen, histiocytes in interstitial connective tissue, and Kupffer cells in the liver (19, 20). On top of that, other organs and tissues in the body also have different types of macrophages that perform different functions and phenotypes. For example, in the intestine, macrophages of different phenotypes will be able to work together to maintain the balance of flora in the gut. Secondary lymphocytes, similar to those in the spleen, contain a large number of macrophages that self-initiate adaptive and antiviral immune responses (16, 21, 22). Unique macrophages reside in immune-privileged sites such as the brain, eye, and testis, which play a central role in tissue remodeling and homeostasis (23–25). Although the developmental origin of tissue macrophages has been widely recognized, there is high functional heterogeneity of macrophages even from the same origin, especially true for macrophage subsets in the cardiovascular system, which are functionally different. In addition, the investigators had performed single cell RNA sequencing against aortic cells from atherosclerotic mice (26). It was found that the expression profile of genes in aortic resident macrophages was analogous to that of aortic resident macrophages in healthy aortic bone marrow cell populations, whereas monocytes, monocyte-derived dendritic cells, and two macrophage populations were almost exclusively present in atherosclerotic aorta, including inflammatory macrophages showing Il1b enrichment and those expressing TREM2 (trigger receptor 2 expressed on bone marrow cells) macrophages showing Trem2 enrichment. The gene expression profile of TREM2-expressing macrophages appears to be similar to that of osteoclasts and may have a regulatory role in calcification in addition to functions in lipid metabolism and catabolism (26, 27).

### 2.2 Macrophage phenotypes and their related regulatory mechanisms

Macrophages exert high plasticity capable of rapidly changing their function *via* a process called polarization, by which macrophages respond to stimuli from the local microenvironment and acquire specific functional phenotypes. Macrophages are typically classified as classically activated, pro-inflammatory, or M1 (28, 29) and vicarious activated, anti-inflammatory, or M2 (30, 31). The specific gene expression program results in the acquisition of the distinct signers on the surface of macrophages, secretion of different cytokines, and metabolic adaptation. For example, unpolarized macrophages in humans are usually labeled with CD14, colony stimulating factor 1 receptor (CSF1R) and CD68, M1 with CD86, CD64, nitric oxide synthase 2 (NOS2), CXCL10, suppressor of cytokine signaling 1 (SOCS1), M2 with CD163, transglutaminase type 2 (TGM2), arginase 1 (ARG1) and so on (32, 33). Meanwhile, M1-type macrophages of mice show low or no expression of CD68, CD64, found in inflammatory zone 1 (Fizz1), chitinase-like protein 3 (Chil3) and other markers are obtained on the surface of M2-type cells (34). More importantly, the balanced polarization of M1/M2 macrophages governs the fate of an organ during inflammation or injury. When the body is exposed to an external infection or autoimmune inflammation severe enough to impact an organ, macrophages exhibit an M1 phenotype to counteract the stimulation of the release of TNF-α, IL-1β, IL-6 and IL-23 (35, 36). However, if the M1 stage continues, it will lead to tissue damage. Therefore, M2 macrophages secrete large amounts of IL-10 and TGF-β to suppress inflammatory responses, promote tissue repair, remodeling, angiogenesis, and maintain homeostasis (37).

**Figure 1:**
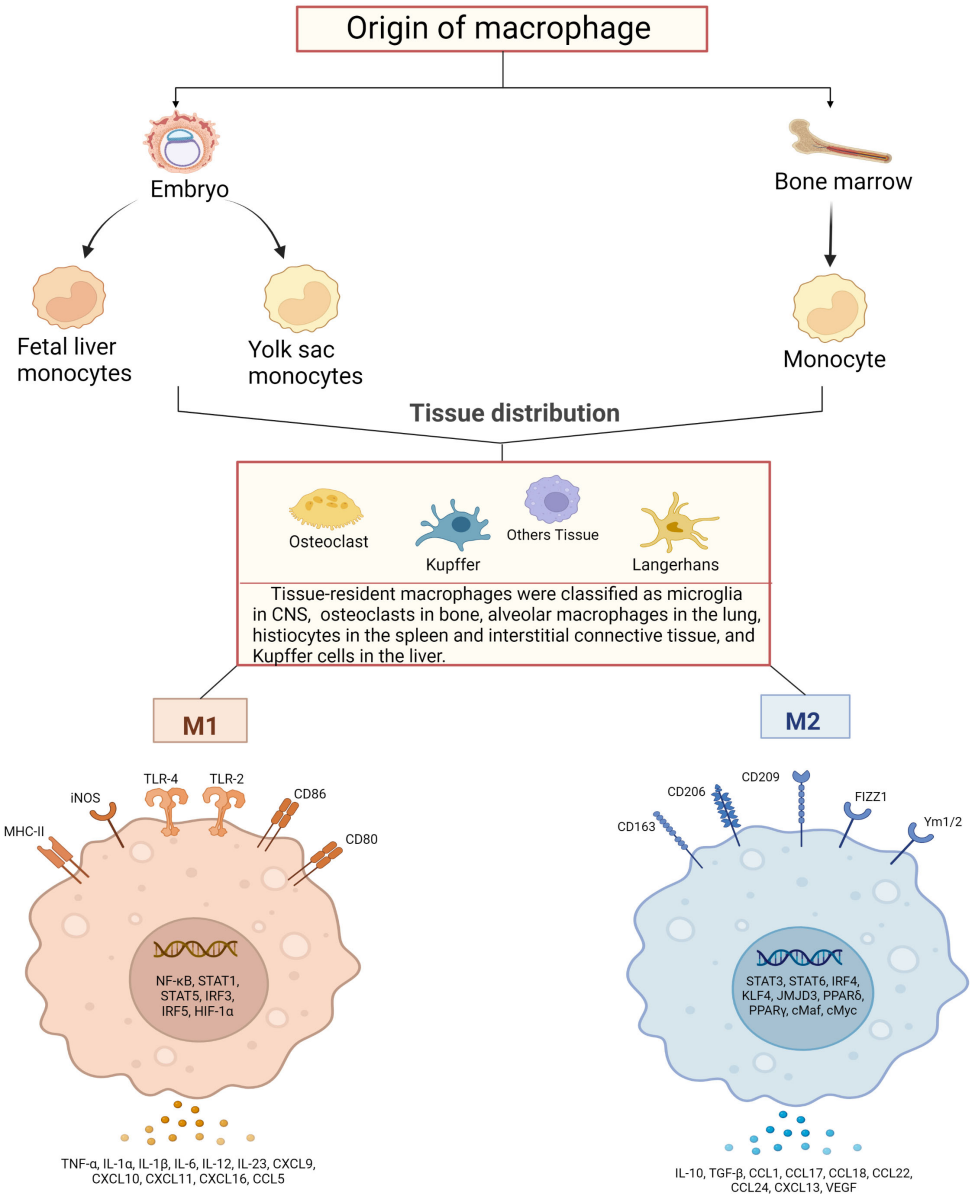
Schematic diagram of macrophage sources, distribution and their activation. Macrophages are derived from bone marrow and embryonic stem cells, and some bone marrow-derived monocytes subsequently flow into tissues to develop into tissue-resident macrophages together with embryonic hepatocytes and yolk sac cells, and specifically differentiate into different functional macrophages at different sites. They have in common that both can polarize into pro-inflammatory (M1) and anti-inflammatory (M2) phenotypes. M1-type macrophages are activated by TLRs ligands and involve several transcription factors, such as NF-κB, STAT1, STAT5, IRF3, IRF5 and HIF-1α, while releasing pro-inflammatory and chemokines including TNF-α, IL-1α, IL-1β M2 macrophages activate transcription factors including STAT3, STAT6, IRF4, KLF4, JMJD3, PPARδ, PPARγ and release anti-inflammatory substances, cytokines and chemokines including IL-10, TGF-β, CCL17, CCL18 and CCL22, which promote tissue repair and regeneration, immunosuppression.

The macrophage polarization has been orchestrated and fine-tuned by key mediators in the milieu. Interferon has long been recognized as a signal sensor for the initiation of inflammatory macrophages and plays a crucial role in the induction of M2 macrophage activation in particular (38, 39). TNF receptor associated factor 6 (TRAF6) is an important signaling node in the Toll-like receptor pathway, which can initiate the transcription of inflammation-related target genes (40). However, members of the CCAAT enhancer binding protein family and signal transducer and activator of transcription (STAT) family have been identified as key mediators of these responses (41, 42). In addition, the regulators of lipid metabolism peroxisome proliferator-activated receptor γ, circular RNAs, microRNAs, and long noncoding RNAs have been shown to be key regulators of macrophage polarization in both *in vitro* and *in vivo* models (43–46) (**Figure 1**).

### 2.3 Evidence to show macrophage recruitment promotes PAH

A distinctive feature of vascular remodeling associated with pulmonary hypertension is the accumulation of macrophages in the perivascular/epithelial membrane. In the PAH setting, macrophages appear to be one of the major cells responsible for causing inflammation in the regional lung (47). Out of the inflammatory cells implicated in PAH, macrophages usually influence the severity and progression of the disease. A variety of studies in experimental animal models have shown that perivascular macrophages perform a central role in the vascular remodeling associated with PAH (48, 49). In a mouse model of chronic thromboembolic pulmonary hypertension, significant aggregation of macrophages expressing specific markers was seen in high-volume arterial vasculature. In the rat model, macrophages were increased in number compared to controls and IL-6, IL-10 secretion was increased in the lungs (50). Also, the same results were found in diseased vessels of patients with PAH caused by left heart disease, whereby there was a significant increase in the number of macrophages around the vessels. In addition, macrophages in the vascular epithelium remained the most pronounced inflammatory cells in the vessel wall in patients with end-stage PAH who underwent lung transplantation (51).

Studies have shown that whole lung samples show up-regulation of pro-inflammatory M1 and alternatively activated M2 macrophage markers in a hypoxia-induced PAH model (52). A recent study showed that the inhibition of M2-type macrophage activation by Donepezil in monocrotaline (MCT)-treated rats could effectively reduce the proliferation of PASMCs and improve pulmonary vascular remodeling (53). To further verify the presence of altered macrophage polarization in PAH patients, it was investigated that an M1/M2 imbalance was observed between macrophage low (MacLow) bone marrow-derived macrophages in men and monocyte-derived macrophages in PAH patients under doxycycline and interleukin-4 (IL-4) stimulation. In addition, MacLow-derived alveolar macrophages exhibited characteristic differences in polarization and diphtheria toxin A-chain expression in response to doxycycline stimulation. This implies that immune cells are involved in this paradigm and that targeting the imbalance in macrophage numbers may provide a future therapeutic option (54).

To further determine the crosstalk that exists between macrophages and pulmonary artery SMC, Researchers showed that macrophage derivatives such as platelet-derived growth factor-belisa (PDGF-B) are essential for pathological SMC expansion in PAH (55, 56). They found that after hypoxic exposure, *Pdgfb* mRNA was up-regulated in mouse macrophages and *Pdgfb* expression was down-regulated in mouse macrophages carrying *hypoxia-inducible factor 1α* (*hif-1α*), *hypoxia-inducible factor 2α* (*hif-2α*) or the *Pdgfb* allele *LysM-Cre* and protected from distal muscularization and PAH. Conversely, *LysM−Crevon-Hippel Lindau^fl/fl^* mice had increased macrophage *Pdgfb* and developed distal muscularization, PH, and right ventricular hypertrophy (RVH) in normoxia (57, 58).

It has also been shown that in hypoxia-induced PAH mouse lungs, expression of tagged C-C chemokine receptor 5 (CCR5) (59), interleukin-1 receptor 1 (IL-1R1) and myeloid differentiation primary response protein 88 (MyD88) is predominantly localized to pulmonary arterial SMC (PASMC), whereas mice with CCR5, IL-1R1 and MyD88 gene disruption and inactivation show PASMC proliferation during hypoxia exposure and perivascular and alveolar macrophage recruitment was reduced, suggesting that PASMC-derived CCR5, IL-1R1 and MyD88 may mediate macrophage recruitment (60, 61), which provides a basis for further exploration of potential targets in the therapeutic process of macrophages and pulmonary hypertension.

### 2.4 Pathological effects of macrophages on other cardiovascular diseases

In addition to the essential point of macrophages in the progression of PAH, macrophages have been reported to play a major role in other cardiovascular diseases. It has been reported that macrophages play a vital role in all stages of atherosclerosis, among which foam cells formed by macrophage lipid metabolism disorders are markers of atherosclerotic (AS) plaque formation (62). There is evidence to suggest that proteolytic cleavage of the macrophage efferocytosis receptor c-Mer tyrosine kinase (MerTK) reduces efferocytosis and promotes plaque necrosis and defective resolution (63). Epidemiological studies have found that blood monocytes of hypertensive patients show obvious pro-inflammatory phenotype, and the content of inflammatory factors in serum is also significantly increased (64). In chronic angiotensin (Ang)II perfusion model, macrophage clearance can significantly prevent blood pressure rise, improve vascular endothelial and smooth muscle cell dysfunction, and reduce vascular reactive oxygen species (ROS) formation (65). In addition, according to the characteristics of macrophages, altered interactions between macrophages and damaged tissues in patients with ischemic heart disease may be a key factor in improving the regenerative potential of the heart. For example, macrophages derived from yolk sacs produce moderate inflammatory reactions and secrete various cytokines, such as chemokine ligand 24 (CCL24) and oncostatin-M (OSM), to promote cardiac recovery after MI and enhance the formation of neovascularization (66, 67). In addition, there are studies based on mouse models showing that resident macrophages prevent arterial stiffing and collagen deposition in the steady state, mainly due to the expression of the hyaluronic acid (HA) receptor LYVE-l on the surface of macrophages, which bind to the HA pericellular matrix of SMCs, thereby regulating SMC collagen expression (68). In conclusion, the function of macrophages varies in cardiovascular diseases, and their pathogenesis may be different.

## 3 Pathological mechanism of PAH

### 3.1 Classical pathological mechanism pathway

The imbalance of vasodilation and contraction caused by early pulmonary vascular endothelial function injury and excessive proliferation of pulmonary SMC is often considered as the main pathological mechanism of PAH. Three classical pathways (69) including prostacyclin pathway, endothelin-1 pathway and nitric oxide pathway, have been identified as the main pathways leading to excessive pulmonary vascular contraction.

Endothelin-1 (ET-1) is a small molecular active substance composed of 21 amino acids. Endothelin mainly exists in the precursor form in tissues and needs to be catalyzed by endothelin convertase to form active endothelin (70). Through bonding to two G protein-coupled receptors (GPCR) on pulmonary SMCs, endothelin-a and endothelin-b (ETA and ETB) receptors, ET-1 promotes the release of Ca^2+^ stored in cells and the opening of voltage-dependent Ca^2+^ channels, resulting in the increase of intracellular Ca^2+^, and further leading to vascular proliferation, hypertrophy, fibrosis, contraction and inflammation (71, 72). In addition, the increased expression levels of ETB on ECs can stimulate the production of nitric oxide (NO) and prostacyclin, thus leading to blood vessel vilation (73).

Ambrisentan, macitentan, and bosentan have been approved as ET-1 receptor antagonists to inhibit the vasoconstrictor activity of ET-1. Although amburecentine has a higher inhibitory effect on ETA, macetan and Bosentan are better choices as dual antagonists of ETA and ETB (74).

ECs are the principal generator of prostacyclin, which is synthesized by arachidonic acid under the action of cyclooxygenase (COX) and prostacyclin synthase (75). Prostacyclin released by ECs binds to GPCR and prostaglandin (IP) receptors on SMCs and activates adenylate cyclase, converting adenosine triphosphate (ATP) into a second messenger, cyclic adenosine phosphate (cAMP). It regulates smooth muscle relaxation and inhibits proliferation by activating cAMP-dependent protein kinase A and cAMP-activated exchange protein. In addition, prostacyclin can reduce platelet aggregation, inhibit smooth muscle cell proliferation, and play an antithrombotic and anti-inflammatory role (76).

Under the action of endothelial nitric oxide synthase in ECs, L-arginine is converted to L-citrulline and a small amount of NO is produced (77). NO spreads to pulmonary vascular SMCs, binds to guanosine guanylyl cyclase (GC), converts guanosine triphosphate (GTP) into cyclic guanosine monophosphate (cGMP), and then activates downstream cGMP-dependent protein kinase (PKG) to decrease myofilament tension, dilate SMCs, produce vasodilation and reduce pulmonary artery pressure (78). Studies have shown that the damage of NO-GC-CGMP-PKG pathway is one of the most important changes in the development of PAH. This is due to the abnormal expression of iNOs in ECs, the decrease of NO production, the decrease of NO bioavailability, the decrease of GC and PKG activities and the increase of type 5 phosphodiesterase activity caused by oxidative stress (79). PAH is caused by a decrease in the production of available vasodilators such as NO (80). Three drugs have been approved for this route, including tadalafil, vardenafil and sildenafil (81).

In view of the above classical pathway, a series of targeted drugs have been approved for clinical treatment of PAH, but these drugs are for symptomatic treatment. There is an urgent need to carry out more in-depth research on the pathogenesis of PAH.

### 3.2 Macrophages mediate PAH by interfering with immunomodulatory mechanisms

Inflammation is a prominent feature of PAH. The synergistic interaction between infiltrating cells and inflammatory cells plays an important role in the pathogenesis of PAH (10). Since macrophages have a vital catalytic role in the inflammatory response, it has been shown that macrophages exacerbate the PAH process by participating in immune homeostasis and by promoting adaptive immune responses during infection. In addition, other immune cells such as T lymphocytes, B cells, DC cells, mast cells and other inflammatory cells all have different roles on the course of PAH (7).

Macrophages are central regulatory cells for T cell activation, which participate in every step of T cell activation. Macrophages can regulate and provide effective costimulatory signals and cytokine secretion for T cell activation. T cells participate in pulmonary vascular remodeling and inflammation in PH by IL, TNF-α and interferon-γ (IFN-γ) (82). In addition, injection of T cells into rats for immune reconstruction can inhibit the inflammatory reaction of pulmonary artery wall and the apoptosis of ECs, thus blocking the occurrence of PH (83), suggesting that Treg cells may play an important role in inhibiting the occurrence and development of PAH.

Studies have reported that B cells promote monocytes to infiltrate into inflammatory sites of pulmonary vessels by secreting CCL7, and monocytes further differentiate into macrophages in inflammatory tissues (84, 85). The infiltration of B lymphocytes and the generation of ectopic lymphoid tissue (tertiary lymphoid tissue) can be seen around the pulmonary vessels of PAH lesions, as well as the deposition of immunoglobulin and complement molecules (86). In addition, a significant increase in the blood in patients with PAH of autoantibodies, including anti-endothelial cell antibodies, fibroblasts, resisting ribonucleoprotein antibodies and anti-topoisomerase I, these antibodies can be combined with vascular ECs and induce its apoptosis, inflammation response, promote the proliferation of vascular SMCs, resulting in the formation of the PAH (9).

Monocytes are derived from hematopoietic stem cells in bone marrow. When monocytes migrate into tissues and organs of the whole body, they develop into mature macrophages, which perform biological functions such as presenting antigens and regulating immunity (14). Under the stimulation of granulocyte-macrophage colony stimulating factor (granulocyte-macrophagecolony-stimulatingfactor, GM-CSF) and IL-4 *in vitro*, monocytes can differentiate into dendritic cells (DCs) (87). Promote the initiation and differentiation of T cell response and participate in adaptive immune response (88). It was found a large number of immature DCs clustered near the pulmonary tissue of IPAH patients and animal models, and the number was positively correlated with the severity of the disease (89). However, the quantity of mature bone marrow-derived DCs in the peripheral blood of IPAH patients is reduced and accompanied by functional defects. Studies have shown that DCs produce chemokine (C-X3-C motif) ligand 1 (CX3CL1) (90), which promotes the proliferation of SMCs in PAH. Therefore, some scholars believe that DCs may be involved in the pathogenesis of PAH.

In addition, many studies found that many studies have found significantly elevated mast cells count and function in PAH patients and animal models (91). It was found that mast cells B cell axis are involved in PAH vascular remodeling. Activated mast cells can produce a large amount of IL-6, which is directly involved in the vascular remodeling process on the one hand, and immunoglobulin and autoantibody production on the other hand (92). Blocking IL-6 or inhibiting mast cells activation can significantly reduce the generation of B lymphocytes, and alleviate the remodeling and hemodynamics of PAH vessels (7). Similarly, inhibiting B-lymphocyte activity or knockout B-lymphocyte activity in PAH rats decreased trypsin, vascular endothelial growth factor, and leukotriene 4 (LTE 4) levels in PAH patients (93, 94). It is suggested that the mast cell IL-6-B lymphocyte axis plays an important role in the pathogenesis of PAH.

**Figure 2:**
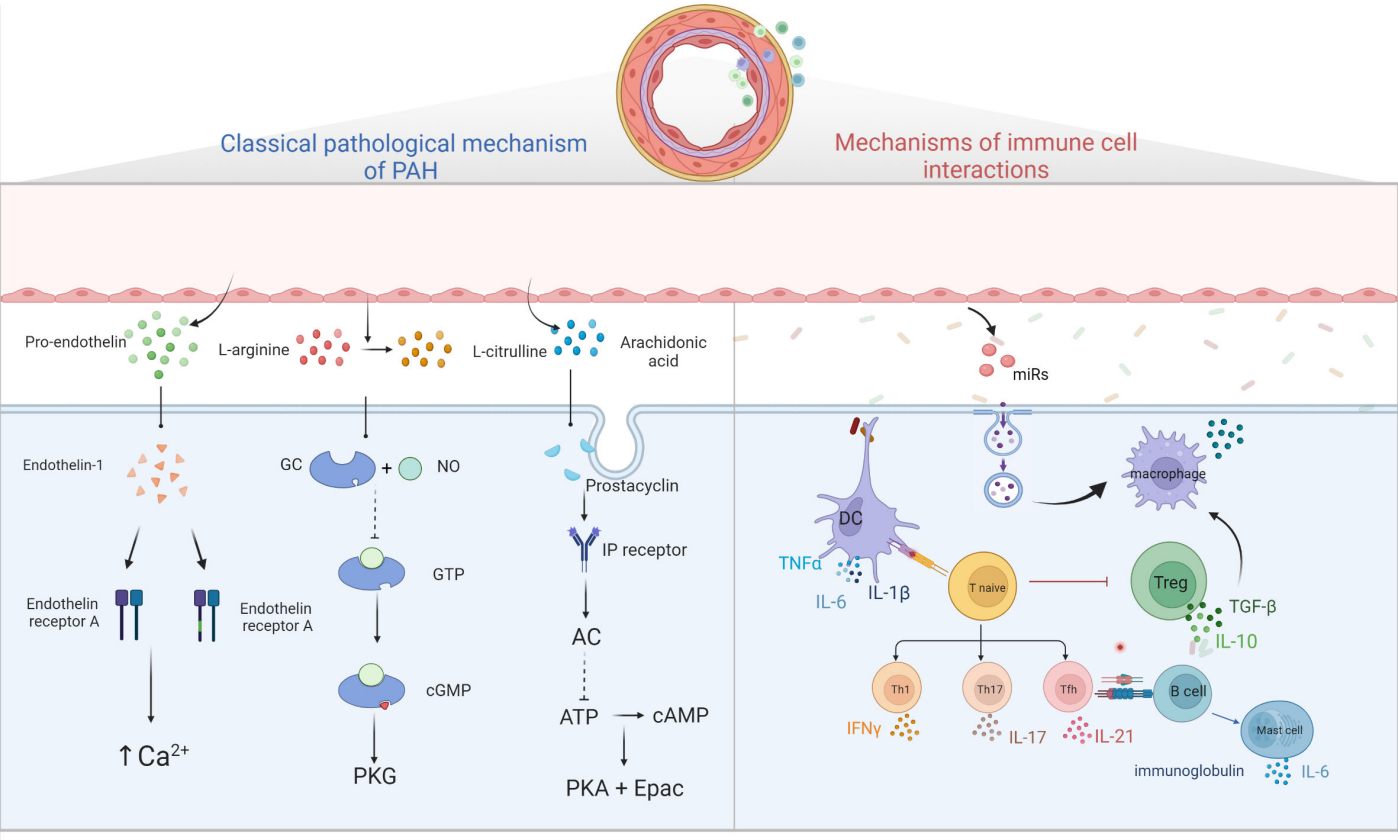
Diagram of PAH pathological mechanism. Three classical pathways; ET-1 binds to ETA and ETB receptors to promote intracellular Ca^2+^ release and Ca^2+^ channel opening, leading to increased intracellular Ca^2+^ and vascular remodeling; prostacyclin binds to GPCR and IP receptors, activating adenylate cyclase, converting ATP to cAMP and inhibiting cell proliferation by activating PKA and Epac; NO binds to GC, converting GTP to cGMP, thereby activating downstream PKG and causing vascular diastole. Immune cells and pulmonary hypertension, DCs stimulate T cell activation, T cells participate in pulmonary vascular remodeling by producing IL, TNF-α, IFN-γ, B cells participate in pulmonary vascular remodeling by over-secreting antibodies, cytokines, etc. Mast cells participate in the development of PAH by producing large amounts of IL-6, while reducing IL-6 production or inhibiting mast cell activation can reduce B lymphocyte production and can alleviate PAH. EC damages will release microvesicles, which contain miRs, and others, these MVs will stimulate macrophages to secrete cytokine such as TGF-β, and then stimulate PASMC proliferation.

The basic role of the immune system in the development of PAH has been paid more and more attention. PAH is no longer considered to be caused only by dynamic vasoconstriction. Inflammation mediates the vascular remodeling of PAH. Although the mechanism is still unclear, the basic role of the immune system in the pathogenesis has been accepted (**Figure 2**).

## 4 Macrophage microenvironment determines the function of macrophages in PAH

**Figure 3:**
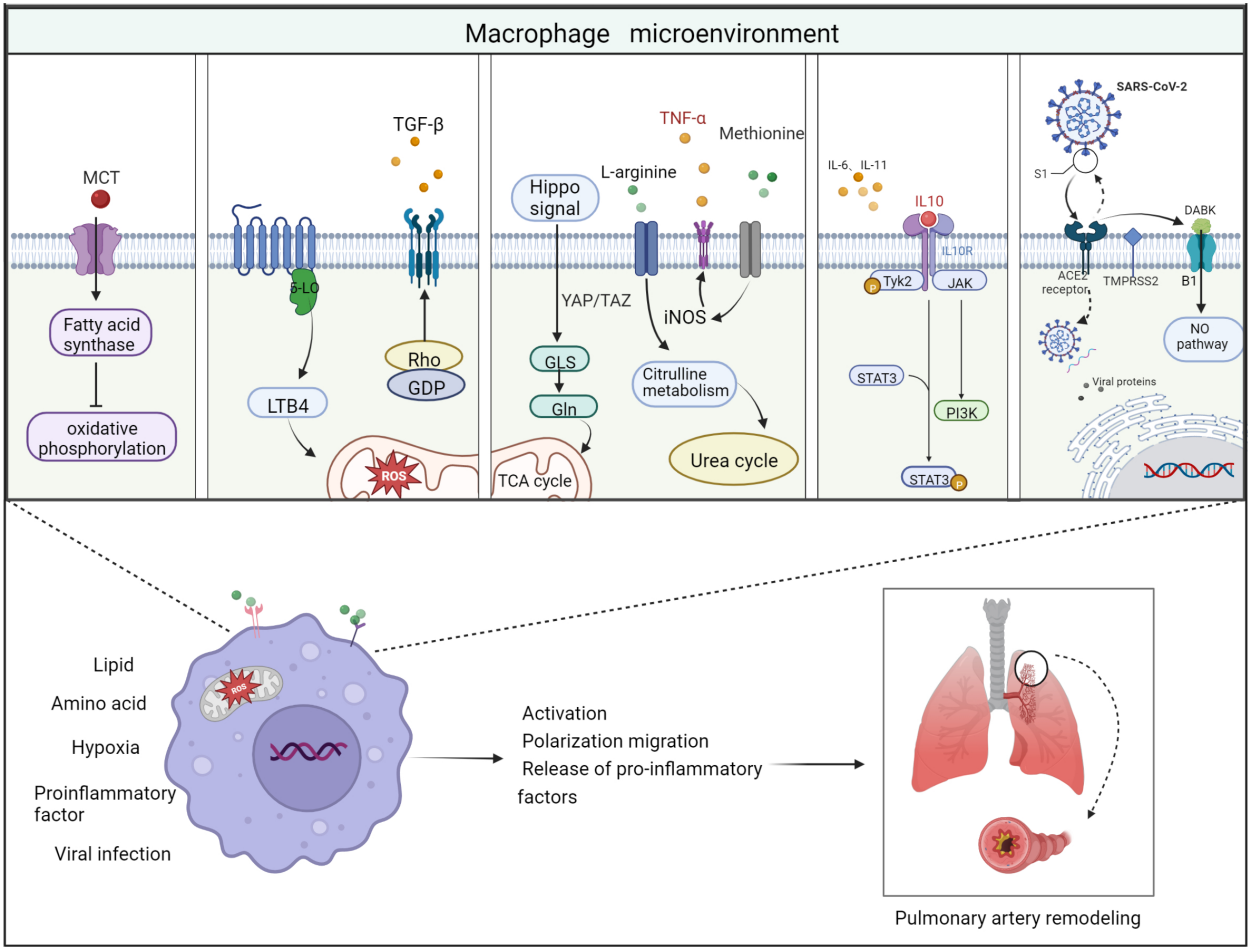
Schematic diagram of the 5 important factors affecting the macrophage microenvironment. Lipid metabolism: monocrotaline induces an increase in body fatty acids and a decrease in fatty acid oxidation. Hypoxic environment: 5-lipoxygenase (5-LO) activation metabolizes leukotriene B4 (LTB4) to exacerbate ROS toxicity in mitochondria; Rho kinase signaling pathway activates TGF-β and causes vasoconstriction. Amino acid metabolism: two related transcriptional co-activators of Hippo signaling pathway, YAP/TAZ, can activate GLS enzymes and promote glutamine metabolism and TCA cycle disorder; arginine can generate citrulline in response to iNOS to participate in urea cycle and promote smooth muscle cell proliferation; methionine can induce elevated iNOS activity and increased TNF-α release, thus causing macrophages to M1 direction of polarization. Pro-inflammatory and anti-inflammatory factors: Inflammatory factors and chemokines secreted by immune cells are involved in the regulation of their respective inflammatory pathways, thus promoting or suppressing the inflammatory response. For example, IL-10 activates JAK1 and TYK2 phosphorylation and thereby activates the STAT3 pathway. Viral infection: Angiotensin-converting enzyme-2 (ACE2) receptor mediates the entry of SARS-CoV-2 virus *via* transmembrane protease serine 2 (TMPRSS2). On the one hand, the increase in free DABK content in the presence of decreased ACE2 function activates B1 receptors and also induces NO pathways to trigger inflammation; on the other hand, the virus attacks the body’s immune function through DNA replication, thus aggravating the body’s immune deficiency. Thus, these factors affecting the macrophage microenvironment can worsen the PAH process when they are deregulated.

Pulmonary vascular inflammatory microenvironment includes intracellular microenvironment and extracellular microenvironment, which are specifically manifested in endothelial cells, fibroblasts, infiltrating immune cells, secreting products of corresponding cells and extracellular matrix composition (95). Macrophages, as a functionally heterogeneous cell population, are subject to changes in phenotype and function depending on the microenvironment in which they are located. In the immunosuppressive microenvironment of pulmonary vascular infiltration, factors such as imbalance of extracellular ion homeostasis, hypoxia, increased reactive oxygen species, and low PH stimulate macrophages to have intracellular metabolic disorders and abnormal transcription. Disturbances in intracellular metabolism can lead to abnormal activation of macrophages thereby disrupting the M1/M2 phenotype balance. This phenomenon leads to an abnormal transformation of the macrophage phenotype with excessive release of some chemokines or growth factors. These growth factors or chemokines accelerate the proliferation rate of pulmonary artery smooth muscle cells and the mesenchymal transformation of endothelial cells (**Figure 3**).

### 4.1 Lipid metabolism

It has been previously shown that disorders of lipid metabolism can cause deterioration of PAH (10). In this process, lipid metabolism is also involved in macrophage activation (96). The synthesis and storage of triglycerides is increased in M1 macrophages in response to stimulation by lipopolysaccharide (LPS) and other pro-inflammatory factors, while M2 macrophages use fatty acid (FA) oxidation for energy supply. FAs mainly exist in blood or other extracellular fluid in the form of lipoproteins, which are covalently linked with glycerol to form triglycerides and phospholipids. Macrophages acquire FAs from lipoproteins in two ways. One is endocytosis of lipoproteins. Macrophages can only ingest metabolized triglyceride-rich lipoproteins. The second one is free FAs released by extracellular hydrolysis of glyceric acid in lipoproteins. Lipoprotein lipase (LPL) secreted by macrophages is the key enzyme for the extracellular release of FAs from triglyceride-rich lipoproteins.

An animal study found that MCT induced an increase in fatty acid synthase expression and activity in lung tissue of rats with PH. The increase of CD36 expression levels in M1-type macrophages in the right ventricle, leading to an increase in body fatty acid levels and a decrease in fatty acid oxidation (97), while inhibition of fatty acid synthesis up-regulated oxidative phosphorylation, which in turn inhibited PASMC proliferation and enhanced pulmonary vascular remodeling and right ventricular hypertrophy (98).

Furthermore, it has been well known that a bioactive lipid site 1 protease (S1P) plays an important regulatory role in vasoconstriction, proliferation, fibrosis and vascular inflammation (99). S1P is a key regulator of several cardiovascular and pulmonary pathophysiological processes, including PAH (100). Recently endothelial monocyte activating polypeptide-II (EMAP II) was found to produce S1P in a two-pronged manner by triggering bimodal phosphorylation of sphingomyelin, a common and coherent upstream signal in inflammatory macrophages and SMCs. Sphingomyelin regulates phosphorylation, transcriptional regulation and translocation of sphingosine kinase 1 in these cells (101). This suggests that EMAP II functions specifically to initiate downstream cellular pathophysiology, providing a reference for the discovery of new potential therapeutic targets for PAH.

### 4.2 Amino acid metabolism

There was earlier evidence to show transient glutamine depletion in human cell lines leads to disruption of the TCA cycle and autophagy, while mTOR signaling activation is usually activated under more severe glutamine deprivation (102), which in turn leads to the expression and secretion of IL-8 and other chemokines. Researchers found that α-ketoglutarate, which is produced by glutamine decomposition plays a special role in M2 activation of macrophages (103). This M2 promotion mechanism is regulated by high α-ketoglutarate/succinate ratio, while its low ratio enhances the pro-inflammatory phenotype of classically activated macrophages (M1 type).

In contrast, the role of glutamine metabolism in the development of PAH, particularly glutaminase (GLS) as the key enzyme that initiates the glutamine hydrolysis pathway, has attracted more and more attention worldwide in recent years (104). Current studies have demonstrated that pro-inflammatory factors secreted by inflammatory cells can cause extracellular matrix (ECM) remodeling and stiffness, leading to pulmonary sclerosis, which is an important component in the pathogenesis of PAH (105, 106). However, it has been found that pulmonary vascular sclerosis activates two relevant transcriptional co-activators of the Hippo signaling pathway, YAP/TAZ, early in PAH, and subsequently YAP/TAZ (Yes-Associated Protein/Transcriptional co-activator with PDZ-binding motif) activates glutaminase enzymes that promote glutamine metabolism and mesenchymal responses, leading to changes in the extracellular environment, such as macrophage recruitment, which in turn leads to PAH (107, 108).

In addition, in cardiovascular diseases, including PAH, L-arginine plays a key role in the initiation of intracellular signaling pathways that trigger inflammatory responses in macrophages. Extracellular L-arginine is essential for the activation of all mitogen-activated protein kinases (JNK1/2, ERK1/2, and p38) and significantly accelerates the stimulation of macrophages by LPS (109). In addition, arginine can generate sterilizing NO and citrulline under the action of iNOS, among which citrulline can participate in the urea cycle and promote smooth muscle cell proliferation and division (110). M2-type macrophages can induce ARG-1 to catalyze arginine metabolism to produce ornithine and urine, which can promote collagen synthesis, pulmonary artery smooth muscle proliferation and tissue remodeling.

### 4.3 Hypoxia environment

The hypoxia-induced PH model in mice shows that macrophages are activated and inflammatory markers are expressed in the early and transient stages of anoxic inflammation (48). Hypoxia stimulates alveolar macrophages to differentiate into M2 phenotype, which is required for vascular remodeling and subsequent establishment of PAH (111, 112). In animal models of chronic hypoxic induced PH, monocytes accumulate around the blood vessels (113). These cells expressing α-SMA protein promote cell proliferation through the production of type I collagen (114). However, liposomes including chlorophosphate or gadolinium trichloride prevent pulmonary vascular remodeling by the reduced production of collagen, fibronectin, and tenascin-C production and accumulation of myofibroblasts (115, 116). Studies have shown that reduction of alveolar macrophages attenuated hypoxia-induced PH, emphasizing the role of pulmonary macrophages in the pathogenesis of PAH (54). Hypoxia M2 macrophage supernatant can promote the proliferation of PASMCs, while carbon monoxide can inhibit this early inflammatory response and improve macrophage infiltration and cytokine production (111, 117). Leukotriene B4 (LTB4) derived from macrophages promotes endothelial injury and proliferation of PASMC (118). Flow cytometry showed that hypoxia-induced PH in mice resulted in the recruitment of circulating classical monocytes into the lungs to become interstitial macrophages, which expressed platelet spondin-1, activated TGF-β by increasing Rho kinase signaling pathway, and caused vasoconstriction (119).

### 4.4 Pro-inflammatory and anti-inflammatory factors

Studies have shown that the body will activate acute inflammation when stimulated by undergoing ventricular shunts or chronic inflammation, especially bone marrow inflammation, during which macrophages adopt a typically activating M1 phenotype to drive the liberation of inflammatory mediators (120). However, this spontaneous immune response mechanism, if uncontrolled, will further deepen tissue damage, while the shift to an anti-inflammatory M2 phenotype helps to facilitate wound healing and tissue repair. Polarization of M1 and M2 phenotypes is a specific response of the body to the outside world, and the dynamic balance between them can inhibit further tissue infection and maintain immune regulatory balance. Studies have shown that the pro-inflammatory cytokines IL-1β, IL-6, IL-12 and TNF-α, produced by monocytes and many other cell types, are implicated in the pathogenesis of primary PAH (PPAH) (121, 122). IL-6 is derived from epithelial cells and SMCs (123), and IL-1β and TNF-α have the ability to induce proliferation of fibroblasts and SMCs (124, 125) and promote thrombosis. Thus, these cytokines may affect the functional architecture of the fibroblasts in the outer layer of the pulmonary vasculature, the SMCs in the middle layer or the ECs in the inner layer, and the disrupted barrier will trigger serious cardiovascular diseases such as microthrombotic lesions and PPAH. In addition, studies have shown that PAH induces PASMC proliferation *in vitro* by distorting the M1/M2 ratio and releasing IL-6 (123). Similarly, MDMs in PAH patients after IL-4 stimulation exhibit depolarization toward the subtype of M2 with a corresponding damage response (126, 127). Interleukin 18 (Il-18), which is mainly secreted by macrophages, is closely associated with the IL-1 family of cytokines and similarly stimulates various pro-inflammatory changes at the target site, including activation of immune effector T cells and an indirect increase in the interferon content, along with positive feedback on the secretion of surface proteins and chemokines from target cell adhesion molecules. More compelling evidence has shown that compared with healthy controls (128), patients with PAH have elevated plasma IL-18 protein, and overexpression of IL-18 in the lung leads to mild dilation of PAH and RV. However, genetic ablation of IL-18 does not reduce hypoxia-induced PAH and RV hypertrophy (129), suggesting that IL-18 may be a modifier of the disease (130).

In addition, chemokines, as pro-inflammatory cytokines that induce cells of the immune system to enter the infection site during the immune response, has a central role to play in the process of PAH. In general, chemokines accumulate in the intimal layer of blood vessels in the form of lipoprotein particles, especially at arterial branches and bends, which are particularly prone to local endothelial cell dysfunction. Stored lipoproteins are modified by various mechanisms, including oxidation, enzymatic processing, demethylation and aggregation, leading to inflammation and activation of surrounding ECs. Activated ECs, in turn, launch of a large number of chemotactic cytokines that cause circulating monocytes in the blood to be transferred to the intima and surrounding spaces of the arterial vessels, where they eventually reside and polarize into monocyte-derived macrophages (131). These macrophages are aggressively consuming lipoprotein rich in esters of cholesterol and subsequently become “foam cells”. Although macrophage uptake of lipoproteins appears to be beneficial, these “foam cells” exacerbate disease by secreting pro-inflammatory mediators, including cytokines and ROS, and ultimately leading to death through necrosis or apoptosis. Studies have shown that CCR4 shows a more obvious M2 activation, as demonstrated by the elevated representation of the archetypal M2 markers ARG1 and FIZZ1 (132–134). Chemokine C-X-C motif ligand 4 (CXCL4/PF-4), a platelet-derived chemokine (135, 136), has been shown to prevent monocyte apoptosis and promote macrophage differentiation *in vitro* (137). In addition, CCR5 is expressed on pulmonary vascular walls and macrophages, and up-regulated in PAH (59). In human tissues, CCR5 is found in ECs, smooth muscle, and macrophages from patients with PAH and is also up-regulated after chronic hypoxia in rodent models. Mice lacking CCR5 are protected by hypoxic PH, and the proliferation of PASMC is reduced (59).

### 4.5 Viral infection

Researchers prospectively studied monkey immunodeficiency virus-associated PH (SIV-PH) and found that all animals exhibited a similar course of SIV acquisition, with an imbalanced plasma environment that was replaced more by an inflammatory infiltrate. Among the PH animals there was a higher frequency of conversion of tissue-resident M1-type macrophages and a lower frequency of anti-inflammatory M2c-like CD68+ macrophages (138).

In addition, during the SARS-CoV-2 virus pandemic a few years ago, it was shown that the angiotensin-converting enzyme-2 (ACE2) receptor mediates the entry of three coronavirus strains: SARS-CoV, NL63 and SARS-CoV-2, which triggers cardiovascular disease and severe lung injury and exacerbates the PAH process (139). They found that ACE2 removes an amino acid residue from the peptide des-Arg bradykinin (DABK), thereby preventing DABK from binding to the bradykinin receptor B1 receptor. When ACE2 function is reduced in the lung as a result of endotoxin, free DABK increases, which in turn activates the B1 receptor and also interferes with adaptive immunity by activating macrophages and other immune system cells, thereby increasing the secretion of IL-6, IL-10 and other inflammatory factors, thereby exacerbating the PAH process (140). It has also been demonstrated that SARSCoV-2 host cell entry is dependent on the SARS-CoV receptor ACE2 and can be blocked by a clinically proven inhibitor of the cytosolic serine protease TMPRSS2, which is used by SARS-CoV-2 for S-protein initiation. Furthermore, this suggests that antibody responses against SARS-CoV may at least partially protect against SARSCoV-2 infection, thereby slowing the progression of PAH, and also provides key insights for identifying potential targets for antiviral intervention (141, 142).

## 5 Crosstalk between macrophages and other cell types

More and more scientists agree that cross-talks between different cell types including endothelial cells, macrophages, fibroblasts and smooth muscle cells are so important for the development of PAH. In either patients with PAH or experimental models of PH, Acute lung injury, viral or bacterial infections or inflammatory cytokines/chemokines may induce endothelial cell damages, which are an initial event for the development of PAH. Damages of endothelial cells release microvesicles which may include miRNAs, caveolin-1 (143). In some patients, red blood cells may be lysed, and release chemokines/cytokines. These microvesicles and cytokines/chemokines activate macrophages to produce TGF-β. TGF-β stimulates smooth muscle cell proliferation and migration (143, 144).

Studies have shown that macrophages are recruited to the periphery of pulmonary vessels and overexpress HO-1, which may respond to Hb-mediated oxidative stress, as excess Hb contacts pulmonary vascular endothelial cells and triggers endothelial apoptosis (145). It has been found that activated macrophages can increase the production of CSF1 protein in fibroblasts *in vitro* and aggravate the inflammatory response (146). Activated fibroblasts produce CCL2, which attracts macrophages to the fibrotic or damaged area. In addition, the expression of pim-1 proto-oncogene (PIM1) and transcription factor NFATC2 in macrophages can be mediated by fibroblast-derived IL-6. And in any IL-6 responder cell, including fibroblasts, macrophages, endothelial cells, and smooth muscle cells, the enhanced proliferative and anti-apoptotic capacity in vascular walls is also controlled by fibroblast-derived IL-6 (147).

## 6 Conclusion

Over the past years, new breakthroughs have been made in the investigation of the pathogenesis and therapeutic strategies for PAH. In addition to the currently known classical therapeutic pathways including the endothelin pathway, prostacyclin pathway, and NO pathway, more and more scholars are focusing on immune inflammation-related therapeutic pathways. This review further elucidates that synergy between macrophages and immune cells have a key role in the pathogenesis of PAH. The microenvironment of macrophages is altered in response to disturbances in the regulation of lipid or amino acid metabolism, hypoxic and inflammatory infiltrative environments, and infections such as viruses, which drive alterations in macrophage polarization and function, ultimately leading to PAH pathological changes. Although the evidence provided by the current study delivers a persuasive argument that macrophages are instrumental in the PAH process, our empirical understanding of the specific mechanisms and roles of macrophages in the PAH process at different times remains immature, and more studies are warranted to better characterize the mechanisms of function and regulation of diverse macrophage subpopulations under normal and pathological conditions.

## Author contributions

M-QZ, C-CW and X-BP drafted the manuscript and prepared the figures. J-ZS, X-MX and ZW participated in the writing of manuscripts and literature collection. H-DZ, Y-FZ and J-WC participated in the drawing of figures. H-RL, J-WC participated in revision of manuscript. Z-YH, L-LZ and Y-YH proposed the concept, and revised the manuscript. All authors contributed to the article and approved the submitted version.
